# Extracellular vesicles produced by the human commensal gut bacterium *Bacteroides thetaiotaomicron* affect host immune pathways in a cell‐type specific manner that are altered in inflammatory bowel disease

**DOI:** 10.1002/jev2.12189

**Published:** 2022-01-22

**Authors:** Lejla Gul, Dezso Modos, Sonia Fonseca, Matthew Madgwick, John P. Thomas, Padhmanand Sudhakar, Catherine Booth, Régis Stentz, Simon R. Carding, Tamas Korcsmaros

**Affiliations:** ^1^ Earlham Institute, Norwich Norwich UK; ^2^ Gut Microbes and Health Research Programme Quadram Institute Bioscience Norwich UK; ^3^ Department of Gastroenterology Norfolk and Norwich University Hospital Norwich UK; ^4^ KU Leuven Department of Chronic Diseases Metabolism and Ageing Translational Research Centre for Gastrointestinal Disorders (TARGID) Leuven Belgium; ^5^ Core Science Resources Quadram Institute Bioscience Norwich UK; ^6^ Norwich Medical School University of East Anglia Norwich UK

**Keywords:** extracellular vesicles, host‐microbe interactions, single‐cell data analysis, toll‐like receptor pathway, ulcerative colitis

## Abstract

The gastrointestinal (GI) tract harbours a complex microbial community, which contributes to its homeostasis. A disrupted microbiome can cause GI‐related diseases, including inflammatory bowel disease (IBD), therefore identifying host‐microbe interactions is crucial for better understanding gut health. Bacterial extracellular vesicles (BEVs), released into the gut lumen, can cross the mucus layer and access underlying immune cells. To study BEV‐host interactions, we examined the influence of BEVs generated by the gut commensal bacterium, *Bacteroides thetaiotaomicron*, on host immune cells. Single‐cell RNA sequencing data and host‐microbe protein‐protein interaction networks were used to predict the effect of BEVs on dendritic cells, macrophages and monocytes focusing on the Toll‐like receptor (TLR) pathway. We identified biological processes affected in each immune cell type and cell‐type specific processes including myeloid cell differentiation. TLR pathway analysis highlighted that BEV targets differ among cells and between the same cells in healthy versus disease (ulcerative colitis) conditions. The *in silico* findings were validated in BEV‐monocyte co‐cultures demonstrating the requirement for TLR4 and Toll‐interleukin‐1 receptor domain‐containing adaptor protein (TIRAP) in BEV‐elicited NF‐kB activation. This study demonstrates that both cell‐type and health status influence BEV‐host communication. The results and the pipeline could facilitate BEV‐based therapies for the treatment of IBD.

## 1 INTRODUCTION

The human gastrointestinal (GI) tract microbiota consisting of bacteria, viruses, archaea, and eukaryotic microbes, contributes to intestinal homeostasis by communicating with various host cells in the intestinal mucosa. Structural, compositional, and functional alterations of the microbiota (“dysbiosis”) are associated with various GI‐related diseases, including Crohn's disease (CD) and ulcerative colitis (UC), two major forms of inflammatory bowel disease (IBD) (Delday et al., 2019). Dysbiosis in IBD is characterised by a reduction in bacterial diversity (UC) or altered composition (CD) that involves *Bacteroides* and *Firmicutes* species (Kabeerdoss et al., 2015). Despite recent advances in our understanding of IBD pathogenesis, the complex interactions between the dysbiotic gut microbiota and the host mucosa that result in aberrant immune activation and inflammation in the gut, are yet to be defined in detail.

*Bacteroides thetaiotaomicron* (Bt) is a Gram‐negative anaerobe that is a major constituent of the human caecal and colonic microbiota (ScienceDirect Topics, 2021). The administration of Bt in murine models of IBD ameliorates inflammation (Chang et al., 2020; Fábrega et al., 2017) with the anti‐inflammatory effects being at least in part mediated by its production of bacterial extracellular vesicles (BEVs). BEVs are released by both commensal Gram‐negative and Gram‐positive bacteria and have the potential to mediate cross‐kingdom interactions with host cells via the delivery of their contents and cargo to affect host cell physiology and function (Chang et al., 2020). BEVs produced by Gram‐negative bacteria, such as Bt, are small, spherical bilayered structures (20–400 nm) composed of phospholipids, lipopolysaccharides, peptidoglycan, outer membrane proteins, periplasmic contents including proteins, and some inner membrane and cytoplasmic fractions (Chronopoulos & Kalluri, 2020; Schwechheimer & Kuehn, 2015). BEVs can permeate through the sterile mucus layer of the colon to access and transmigrate boundary intestinal epithelial cells through different routes (Jones et al., 2020) enabling them to interact with underlying mucosal immune cells (Cecil et al., 2019; Durant et al., 2020; Hickey et al., 2015; Kaparakis‐Liaskos & Ferrero, 2015; Shen et al., 2012) and the intestinal vasculature which facilitates their wider, systemic dissemination (Durant et al., 2020; Jones et al., 2020; Stentz et al., 2018).

For Gram‐negative bacteria, a defined pathway of interaction with the host immune system is via membrane‐associated molecules, including lipopolysaccharide (LPS) (Matsuura, 2013). Immune cells interact with LPS via their pattern recognition molecules such as Toll‐like receptors (TLRs). LPS consists of three main structural components of diverse functions: lipid anchor (lipid A), core oligosaccharide region, and O‐antigen. Lipid A is the most conserved part of LPS. The core region connects the anchor and antigen units, the O‐antigen is the immunogenic portion of LPS consisting of long polysaccharide chains (Arenas, 2012). The structure of LPS is diverse among bacterial taxa resulting in taxon‐specific immune responses in the host. Bt contains lipooligosaccharides (LOS) which are structurally distinct from the prototypical LPS of *Escherichia coli (E. coli)* (Jacobson et al., 2018). For example, while lipid A is both hexa‐acylated and diphosphorylated in *E. coli*, Bt has penta‐acylated and monophosphorylated lipid A that does not promote proinflammatory responses in immune cells (Jacobson et al., 2018; Steimle et al., 2019).

Host cells acquire and degrade BEVs by several pathways including dynamin‐dependent endocytosis, macropinocytosis, and caveolin‐mediated endocytosis (Jones et al., 2020). BEVs and their protein cargo can trigger intracellular signalling cascades in various immune cells such as dendritic cells (DCs). In the healthy gut, this interaction leads to the production of anti‐inflammatory cytokines (such as IL‐10), whereas in the inflamed gut of IBD patients, this anti‐inflammatory response is lost (Durant et al., 2020). Another recent study showed that Bt BEVs enhance regulatory T cell and helper T cell 1 (Th1) responses, while decreasing the activation of Th2 and Th17 cell (Li et al., 2021). These anti‐inflammatory properties of BEVs have led to their incorporation into probiotic‐based therapeutics in murine models of IBD (Chang et al., 2020; Fábrega et al., 2017). There is a major need for such novel therapeutic strategies as despite the advent of biologic therapies in IBD, ∼25% of patients with UC and up to 75% with CD eventually require surgical intervention. One such strategy being explored is the ability to modulate the host immune system through microbiota‐based therapies (Zhang et al., 2017). Given the ability of Bt BEVs to influence host immune cell signalling they may have untapped therapeutic potential.

However, the effects of Bt BEVs on different host immune cells are poorly understood. Single‐cell transcriptomics (scRNAseq) provides an opportunity to understand how Bt BEVs influence gut mucosal immune cell populations with cell‐type specific resolution. Of particular interest are monocytes, macrophages and DCs, which play key roles in initiating and determining the outcome of local and systemic immune responses to non‐harmful and harmful stimuli (Scott & Mann, 2020), and shaping the immune response in IBD (Steinbach & Plevy, 2014).

Here, we have utilised single‐cell RNAseq datasets in combination with Bt BEV proteomes to develop a computational workflow of the predicted effect of BEVs on immune cells at different stages of their development, in healthy and disease (UC) states. In a proof‐of‐concept study, we experimentally confirm the predicted interaction of BEVs with human monocytes via TLR4.

## 2 MATERIAL & METHODS

### 2.1 Characterisation of Bt BEV proteins

The bacterium Bt VPI‐5482 was grown anaerobically at 37°C with agitation using a magnetic stirrer in Brain Heart Infusion (BHI) medium (Oxoid/Thermo Fisher, Basingstoke, UK) supplemented with 0.5 mg/L haemin. BHI (three independent cultures) was inoculated with an overnight culture of Bt at an initial OD_600_ of 0.05. After 5 h of growth (OD approximately 3.0, early stationary phase), the cells were centrifuged at 5500 g for 45 min at 4°C. The supernatants were filtered through polyethersulfone (PES) membranes (0.22 μm pore‐size) (Sartorius) to remove debris and cells. The sterility of the vesicle‐containing filtrates was confirmed by plating onto BHI–haemin agar. BEVs in the 500 ml filtrates were concentrated by crossflow ultrafiltration (100 kDa MWCO, Vivaflow 50R, Sartorius) to 0.5 ml, diluted by addition of 500 ml of ice‐cold phosphate buffered saline (PBS), pH 7.4, and the suspensions were concentrated again by crossflow filtration to 0.5 ml and filter‐sterilised through a 0.22 μm PES membrane (Sartorius). Following crossflow ultrafiltration, further purification of BEVs was performed by fractionation of the suspension (Durant et al., 2020) by size‐exclusion chromatography using a CL2‐B Sepharose (Sigma‐Aldrich) (120 cm × 1 cm column) in PBS buffer. The absorbance of the fractions was measured at 280 nm and the first fractions corresponding to the first absorbance peak were pooled and concentrated to 1 ml with a Vivaspin 20 centrifugal concentrator (100 kDa molecular weight cut‐off, Sartorius) and filtered through a 0.22 μm PES membrane (Sartorius). Vesicle concentration was determined by Nanoparticle Tracking Analysis (NTA). The BEV suspension was centrifuged (150,000 g at 4°C or 2 h in a Ti70 rotor (Beckman Instruments)), the supernatant removed using a vacuum pump and the vesicle pellets were snap frozen in liquid nitrogen and stored at ‐80°C prior to extraction.

### 2.2 Proteomic analysis

Samples for proteomics analysis consisted of 100 ug of BEV or cell protein extract prepared and labelled at the Bristol University proteomics facility using TMT reagents (10‐Plex format, Isobaric Mass Tagging kit, Thermo Scientific). Labelled samples were pooled and then fractionated using High pH Reverse Phase Liquid Chromatography. The resulting fractions were subjected to nano‐LC MS/MS using an Orbitrap Fusion Tribrid mass spectrometer with an SPS‐MS3 acquisition method. Fragmentation of the isobaric tag released the low molecular mass reporter ions which were used to quantify the peptides. Protein quantitation was based on the median values of multiple peptides identified from the same protein, resulting in highly accurate protein quantitation between samples. The data sets were analysed using the Proteome Discoverer v2.1 software and run against the Bt VPI‐5482 and filtered with a 1% and 5% FDR cut‐off.

### 2.3 Transmission electron microscopy

Samples were visualized using negative staining with TEM. Briefly, 4 μl BEV suspension was adsorbed to plasma‐pretreated carbon‐coated copper EM grids (EM Solutions) for 1 min before wicking off with filter paper and negatively staining with 1% Uranyl Acetate solution (BDH 10288) for 1 min. Grids were air‐dried before analysis using a FEI Talos F200C electron microscope at 36,000x–92,000x magnification with a Gatan Oneview digital camera.

### 2.4 Isolation and characterisation of Bt BEVs for the experimental validation

Bt (strain VPI‐5482) was grown with agitation under anaerobic conditions at 37 °C in 50 ml (three replicates) of brain heart infusion (BHI) broth medium (Oxoid/Thermo Fisher, Basingstoke, UK) supplemented with 0.5 mg/L haemin (Sigma‐Aldrich, St Louis, MO, USA) (BHI–haemin) at 37°C to early stationary phase (OD approximately 2.5). 20 ml of each culture was centrifuged at 5,500 g for 20 min at 4°C and the supernatants vacuum‐filtered through polyethersulfone (PES) membranes (0.22 μm pore‐size) (Sartorius) to remove debris and cells. Supernatants were concentrated by ultrafiltration using Amicon ultra‐15 centrifugal filter units (100 kDa molecular weight cut‐off), the retentate was rinsed twice with 15 ml of PBS (pH 7.4) and concentrated to 150 μl. To separate out BEVs from remaining proteins and lipids, qEVsingle/35 nm columns (Izon) were used to perform SEC according to manufacturer instructions. Fractions containing BEVs were combined and the suspensions were stored at 4°C. The size and concentration of the isolated BEVs was determined using a ZetaView PMX‐220 TWIN instrument according to manufacturer instructions (Particle Metrix GmbH). Aliquots of BEVs suspension were diluted 1000‐ to 20,000‐fold in particle‐free PBS for analysis. Size distribution video data was acquired using the following settings: temperature: 25°C; frames: 60; duration: 2 s; cycles: 2; positions: 11; camera sensitivity: 80 and shutter value: 100. The ZetaView NTA software (version 8.05.12) was used with the following post acquisition settings: minimum brightness: 20; max area: 2000; min area: 5 and trace length: 30.

### 2.5 Single‐cell transcriptomic datasets analysis

A publicly available scRNAseq dataset describing gene expressions in 51 cell‐types from the colon in three conditions (healthy, non‐inflamed UC, and inflamed UC) was analysed by using the average expression of genes (Smillie et al., 2019). From the 51 cell‐type datasets, cycling monocytes, inflammatory monocytes, macrophages, DC1 (healthy mucosa‐related subset) and DC2 (inflammation‐related subset) populations appearing in healthy and non‐inflamed UC conditions were selected for further analysis. Raw data is available on the Single Cell Portal (https://singlecell.broadinstitute.org/) under SCP259 study ID. While the original dataset contains inflamed samples, in order to avoid inflammation‐related bias in cell communication we focused our analysis on non‐inflamed cells from the same UC patients.

Raw scRNAseq data was processed using scripts and parameters by Smillie et al (Smillie et al., 2019) (http://www.github.com/cssmillie/ulcerative_colitis). To discard genes expressed at extremely low levels, we applied a z‐score test based on the method of *Hart et al* (Hart et al., 2013). A gene was considered not to be expressed if its log2 expression value was less than three standard deviations of the mean expressed genes in that cell.

### 2.6 THP‐1 monocyte transcriptomic analysis

Two publicly available bulk RNAseq datasets of the human monocytic cell line THP‐1 were used for experimental validation. Raw counts from GSE132408 (https://www.ncbi.nlm.nih.gov/geo/query/acc.cgi?acc = GSE132408) and GSE157052 (https://www.ncbi.nlm.nih.gov/geo/query/acc.cgi?acc = GSE157052) datasets were normalized using the DESeq2 package in R. Due to the different gene symbols and gene IDs in the datasets, we unified them to gene symbols using Uniprot and used only genes detected in both experiments. We applied the same protocol as for the single cell RNA‐seq datasets: first we log2 transformed the count number and then we used a Z normalisation. We considered a gene expressed if its z‐score was above ‐3 (mean ‐3 standard deviation). We used these Z transformed values for the analysis.

### 2.7 Constructing a host cell‐BEV interactome

We predicted the effect of BEV proteins on different cell‐types based on host‐microbe protein‐protein interaction (PPI) networks using our MicrobioLink pipeline (Andrighetti et al., 2020). The connections were based on experimentally verified domain‐motif interactions from the Eukaryotic Linear Motif (ELM) database (Kumar et al., 2020). It was assumed that a BEV protein containing a domain can bind to a human protein having the corresponding interacting motif within its sequence. First, we downloaded the sequence of BEV and human proteins from the Uniprot database (Consortium, 2019). Then Pfam domains of BEV proteins were predicted by InterProScan and human motifs identified by the ELM database. To avoid large numbers of false‐positive PPIs, a quality filter was applied using IUPred tool (Mészáros et al., 2018) which uses scores based on two methods (IUPred and ANCHOR2) to measure residue‐level energy terms. The energy terms correlate how intrinsically disordered the protein region is. Higher disordered regions are more accessible for the bacterial domain. Two cut‐off values (IUPred > 0.5 and ANCHOR2 > 0.4) were set up to select human motifs which are presented out of globular domains and at an intrinsic disordered protein region (Mészáros et al., 2018).

### 2.8 Functional analysis of BEV target proteins

Functional analysis was performed using the Gene Ontology (GO) database. GO database orders the annotations in a tree‐like structure where parent and child categories are represented in a hierarchical way. GOrilla was used to highlight the enriched biological processes of the BEV targets in different cell‐types (Eden et al., 2009). As a background dataset, all expressed genes were examined in cells facilitating the identification of cell‐type specific functions. An annotation was significantly overrepresented among the Bt targets if the *P*‐value was less than 10^–3^ and the FDR q‐value calculated by Benjamini and Hochberg method was less than 0.05. We used REVIGO to reduce the dimensionality of the annotations, thereby avoiding the overlapping processes that belong to the same function and identify significant differences among functions (Supek et al., 2011). simRel scores were applied to measure the GO semantic similarity. To visualise the functional overlap among cell‐types, InteractiVenn was used (Heberle et al., 2015). Although this analysis is suitable for depicting processes that are specific to a cell‐type or condition due to the large number of BEV interacting proteins in each cell‐type, the output of this analysis focuses mainly on common processes. A more fine‐grained analysis can be achieved by involving gene expression values, and not only the presence or absence of a gene's expression when establishing condition specific differences.

### 2.9 Cell‐type and condition specific TLR pathway modelling

Members of the TLR pathway were derived from the Reactome database due to its high and reliable coverage of associating proteins to pathways. (Jassal et al., 2020). The OmniPath database was used to collect the interactions due to slightly larger coverage of interaction data compared to Reactome (Türei et al., 2016). Signalling in different cell‐types was interpreted by adding the expression values from scRNAseq datasets (monocytes, dendritic cells, macrophage) and bulk RNAseq (THP‐1 cells). To compare the signal flow under different conditions (healthy and non‐inflamed UC), expression values were added to the genes/proteins. We created one network for each cell type to represent both conditions. We avoided using differentially expressed genes because it focuses only on the differences at the gene level and not the pathway of the spreading signal. Therefore, the healthy log2 gene expression was subtracted from the diseased expression value to indicate differences in signal flow in the TLR pathway.

### 2.10 TLR‐signalling in THP1‐Blue cells

THP1‐Blue NF‐κB reporter cell line (Invivogen) was derived from the human THP‐1 monocytic cell line by stable integration of an NF‐κB‐inducible secreted alkaline phosphatase (SEAP) reporter construct. THP1‐Blue cells were cultivated in RPMI‐1640 (Sigma‐Aldrich) supplemented with 10% heat‐inactivated FBS (Biosera), 1% Pen/Strep (Sigma‐Aldrich) and 100 μg/ml Normocin (Invivogen) at 37°C and 5% CO_2_ in a humidified incubator. To maintain selection pressure during cell subculturing, 10 μg/ml blasticidin (Invivogen) was added to the growth medium at every other passage. To identify TLR4 and TIRAP mediated activation THP‐1 cells were seeded in flat‐bottomed 96‐well plates at a density of 5 × 10^5^ cells/ml and incubated with *E. coli* derived LPS (10 ng/ml, Sigma‐Aldrich) 1 h at 37°C. Control cultures were incubated with PBS. In some cases, cells were pre‐treated with the TLR4 inhibitor CLI‐095 (2 μg/ml) (Invivogen) or peptide‐based TIRAP inhibitor (50 μg/ml) (Merck) and incubated for 1.5 h at 37°C and 5% CO_2_ in a humidified incubator. For BEV‐THP‐1 co‐culture cells were incubated for 24 h with different concentrations of BEVs (3 × 10^9^, 3 × 10^8^, and 3 × 10^7^/ml) after which 20 μl of the cell suspension was added to flat‐bottomed 96‐well plates, mixed with 180 μl of Quanti‐Blue (Invivogen) colorimetric assay reagent and incubated for 1 h at 37°C. Secreted alkaline phosphatase (SEAP) levels were quantified by absorbance reading at 620 nm. All incubations were performed in triplicate.

### 2.11 Statistical analysis

Data were subjected to one‐way or two‐way ANOVA followed by Bonferroni's multiple comparison post hoc test using GraphPad Prism 5 software. Statistically significant differences between two mean values were established by adjusted *P*‐value < 0.05. Data are presented as the mean ± standard deviation.

### 2.12 Data availability

Raw scRNAseq data was extracted from Smillie et al. (2019). Bulk transcriptomics for THP‐1 cell line analysis can be found in GEO [GSE132408, GSE157052]. The workflow containing Python and R scripts, input files and results is accessible on GitHub (https://github.com/korcsmarosgroup/BT_BEV_project/).

## 3 RESULTS

### 3.1 The BEV–Immune cell protein interactome

**FIGURE 1:**
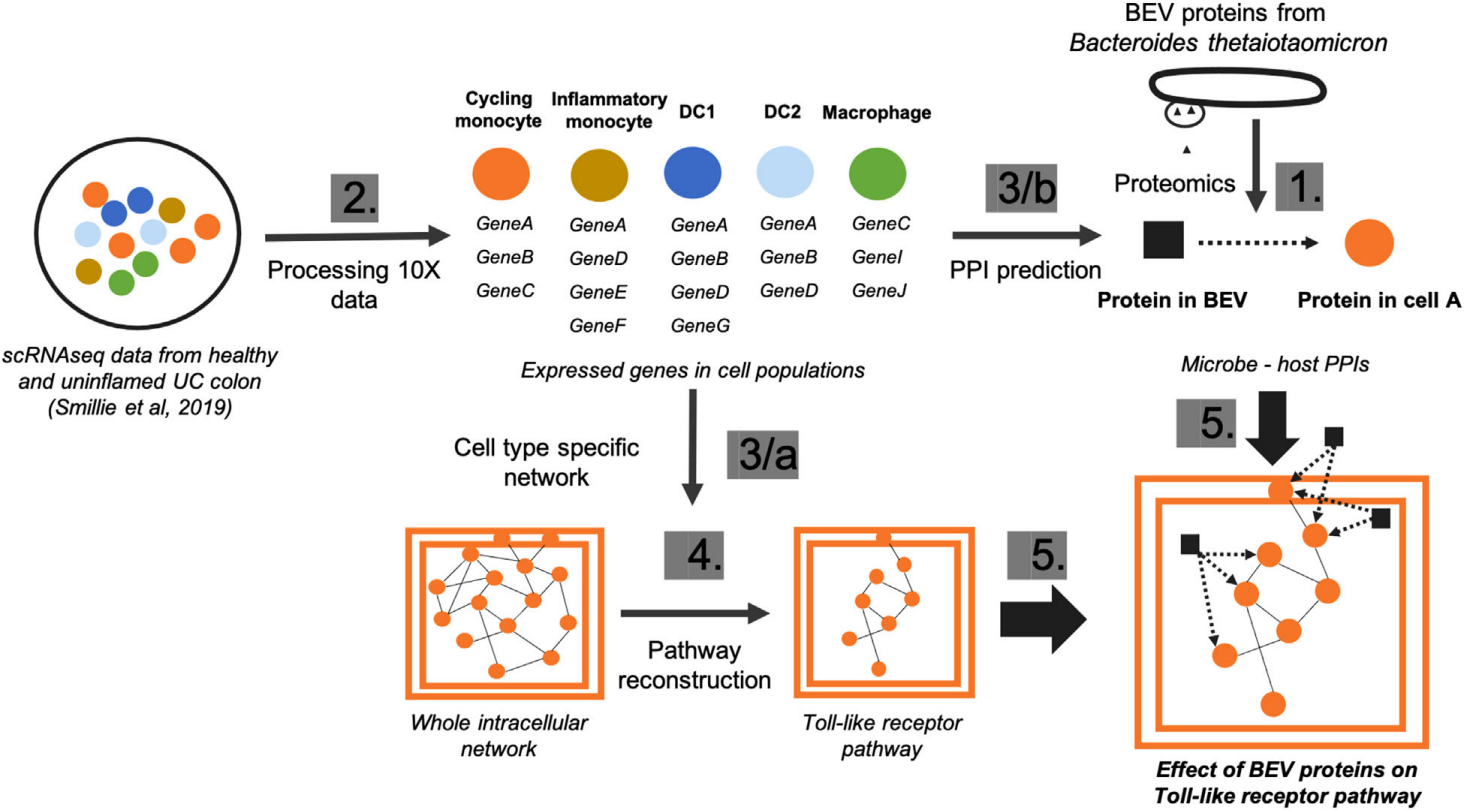
Computational workflow to analyse cell‐type specific effects of BEVs. Numbers indicate the sequence of the main steps: 1, Extraction of BEV proteins from the proteomic dataset 2, Processing the raw single‐cell transcriptomics from human colon 3/a, Creating cell‐type specific network using protein‐protein interactions from OmniPath (Türei et al., 2016) 3/b, Predicting protein‐protein interactions (PPIs) between BEV and host proteins in each cell‐type separately 4, Reconstruction of Toll‐like receptor pathway using Reactome database (Jassal et al., 2020) 5, Combining cell‐specific signalling with BEV targeted human proteins

To analyse the effect of BEV proteins on human cell‐type specific signalling pathways we developed a computational workflow to process single‐cell data, combine information from network resources, and incorporate bioinformatics prediction tools (Figure 1).

**FIGURE 2:**
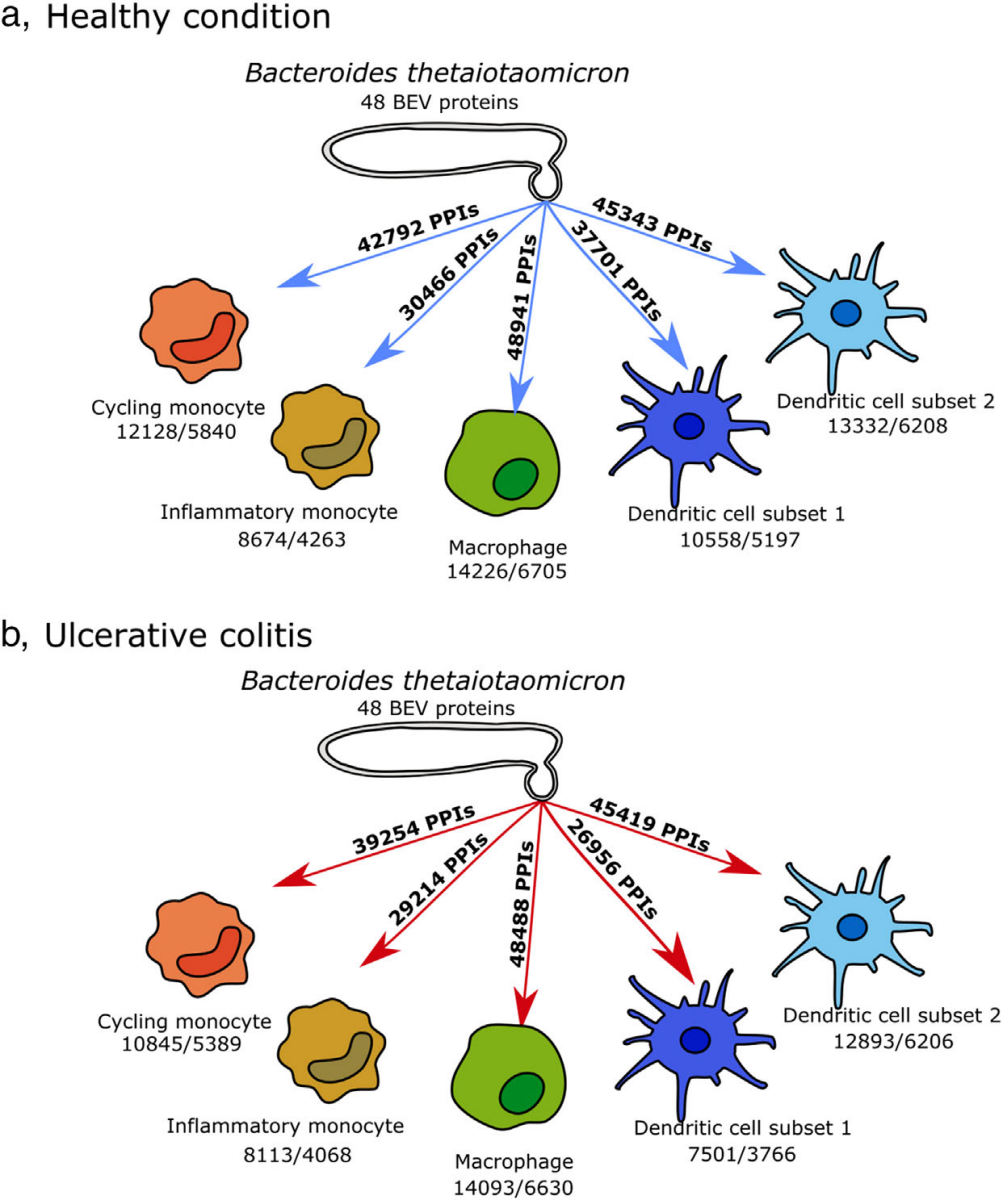
Interactions of 48 BEV proteins with monocytes, macrophages and dendritic cells in healthy (a) and UC (b) conditions. Number of expressed genes/number of interacting proteins are highlighted for each cell‐type

Using this workflow, we identified potential candidates from the proteome of BEVs obtained from a culture of Bt grown in the complex medium BHI, which totalled 2068 proteins. The same proteins were identified in BEVs extracted from the caecum of germ‐free mice monocolonized with Bt (Stentz et al., 2020). TEM was used to determine the purity of BEV preparations (Figure S1). For host cells, scRNAseq data identifying genes expressed in each of five immune cell‐types was used (Figure 2). For the purpose of developing the protein‐protein interaction (PPI) network, we assumed that all of the expressed genes were translated into functional proteins.

BEVs can interact with the host via cell surface receptors and after internalisation, with cytoplasmic receptors. We did not therefore filter host proteins based on their cellular location. Despite the large number of BEV‐human PPIs (Figure 2) the majority of bacterial proteins were hubs indicating they can potentially interact with thousands of host proteins. In total, 48 BEV proteins interact with the host immune cells (Table S1), the majority of which are hydrolases, proteases, and other catabolic enzymes without a specific cleavage site. In terms of individual interactions, five BEV helicase proteins (BT_0831, BT_1154, BT_3303, BT_3844 and BT_3938) were predicted to target the same host protein PAPD5, a non‐canonical poly(A) polymerase whose function is impaired in IBD (Boele et al., 2014; Rammelt et al., 2011).

### 3.2 Functional analysis

Cell‐type specific BEV‐host interactomes are complex due to the large number of proteins and interactions involved. Therefore, a functional analysis based on the GO database was initially carried out to identify the biological processes affected by microbial proteins in healthy (non‐inflamed) and inflamed UC conditions. Most of the over‐represented functions were overlapping among the different cell‐types. However, comparing the cells under different conditions enabled us to identify specific effects of BEV proteins with the unique functions (Table S2 and S3, Figure S2–S, 4).

**FIGURE 3:**
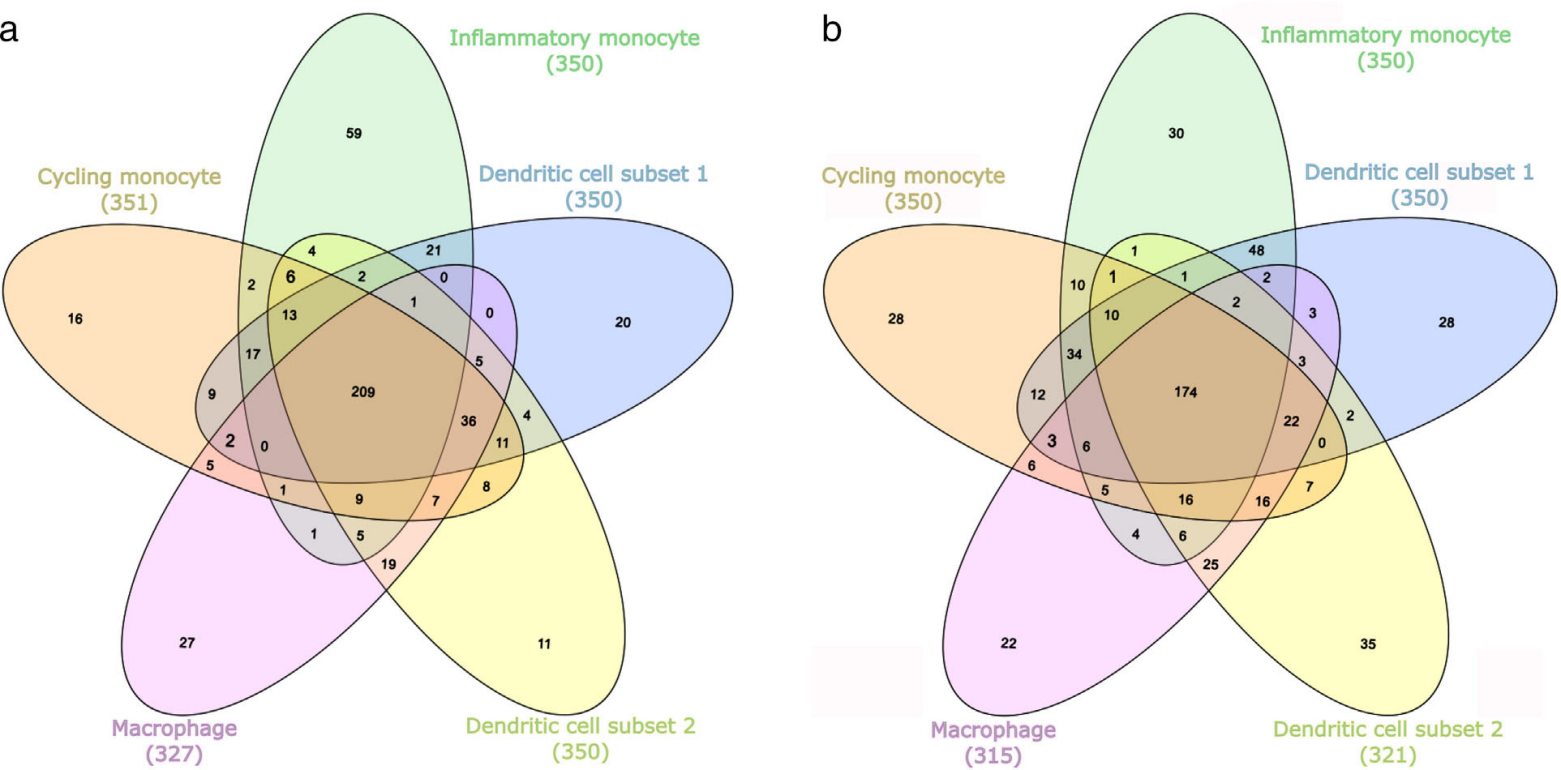
Overlap of biological processes over‐represented in the BEV‐host interactomes corresponding to cell‐types in healthy (a) and uninflamed UC (b) conditions

In the healthy state 209 functions were shared among the five cell‐types containing basic cellular functions, such as chromatin organisation and macromolecule synthesis. Most of the unique processes (59) were found in inflammatory monocytes and were related to the endoplasmic reticulum (ER), apoptosis and myeloid cell differentiation. Counter to these results, in cycling monocytes—in terms of unique functions (16)—cell cycle‐related processes were uncovered. Interestingly, among BEV targets in DC1 cells (20) somatic diversification of immune receptors and B cell apoptosis were uniquely over‐represented. In contrast, negative regulation of myeloid leukocyte mediated immunity and cell differentiation were prominent in DC2 cells (11). Among BEV‐targeted human proteins, the signalling pathways of both the epidermal growth factor (EGF) receptor and the regulation of transforming growth factor beta (TGF‐beta) receptor were affected specifically in macrophages, based on 27 individual processes (Figure 3a).

BEV targets in the non‐inflamed UC state included 174 overlapping processes that play vital roles in cell function. Uniquely over‐represented functions were observed in inflammatory monocytes (30) that were similar in non‐inflamed UC and healthy conditions and included positive regulation of the endoplasmic‐reticulum‐associated protein degradation (ERAD) pathway and intrinsic apoptotic signalling pathways. Among the 28 cycling monocyte‐related annotations, similarly to the healthy condition, the cell cycle associated proteins were overrepresented. Here, we also found the negative regulation of G1/S phase transition overrepresented. Other targeted human proteins identified in this study are involved in the regulation of DNA repair and cyclin‐dependent protein kinase activity, positive regulation of protein ubiquitination, and signal transduction by p53 class mediator. Whereas BEV proteins affected cell‐cycle processes in DC2 (35), target proteins in DC1 (28) related to vesicle fusion, negative regulation of apoptotic signalling pathways, and the intracellular steroid hormone receptor signalling pathway. Among the 22 unique processes in macrophages, regulation of RAS protein signal transduction, base‐excision repair, and diverse histone modification steps were identified (Figure 3b).

In both conditions, macromolecule metabolism, DNA‐related processes, and RNA‐related processes were affected in all five cell‐types by BEVs. Additionally, endoplasmic reticulum (ER)‐stress response related processes and vesicle organisation and transport were influenced by BEVs in most cell‐types.

### 3.3 Effect of BEV proteins on TLR pathway in dendritic cells, monocytes and macrophages in healthy and UC conditions

**FIGURE 4:**
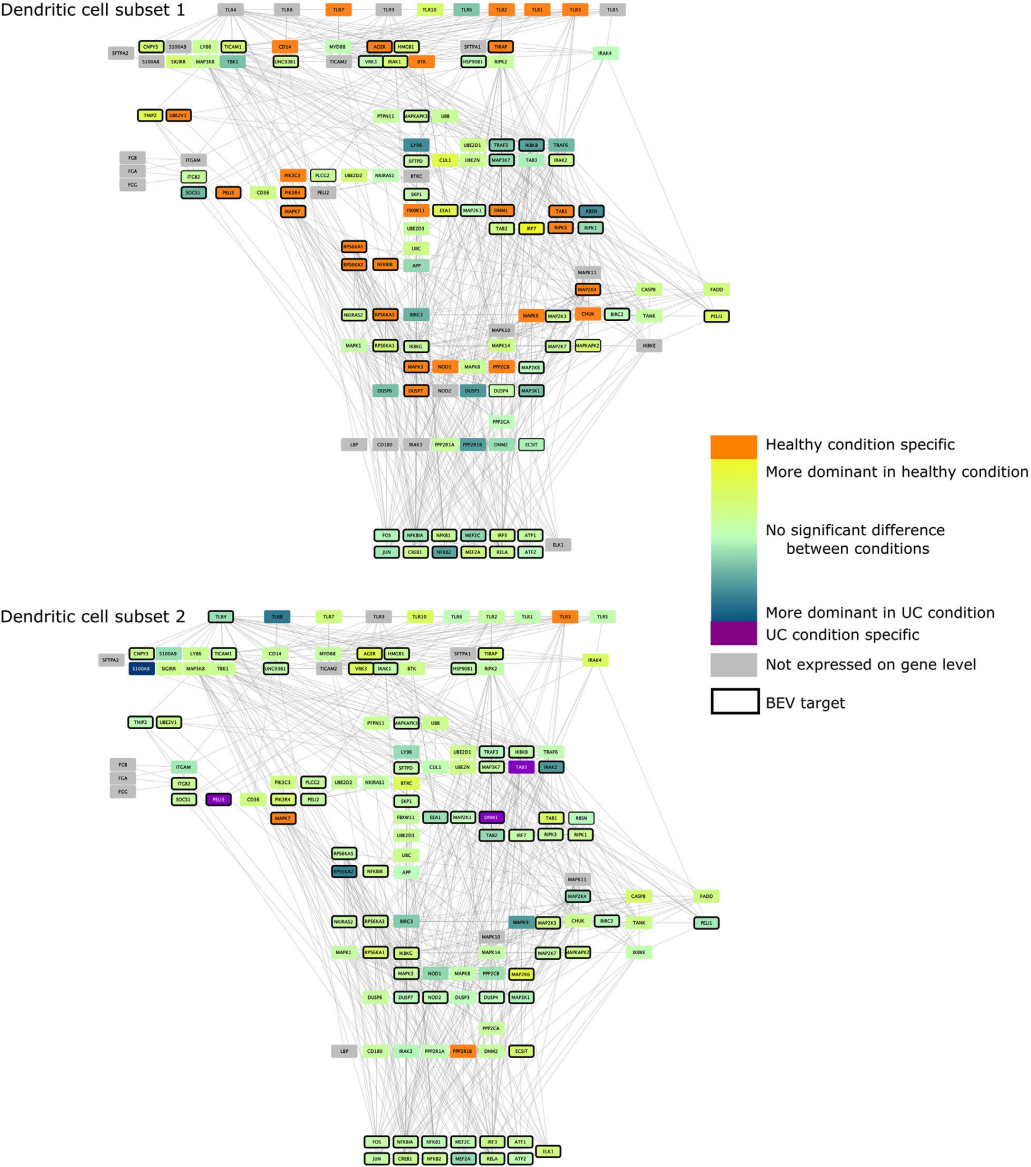
TLR pathway in DCs. Edges between nodes represent protein‐protein interactions. Figures have been created with Cytoscape (Shannon et al., 2003)

**FIGURE 5:**
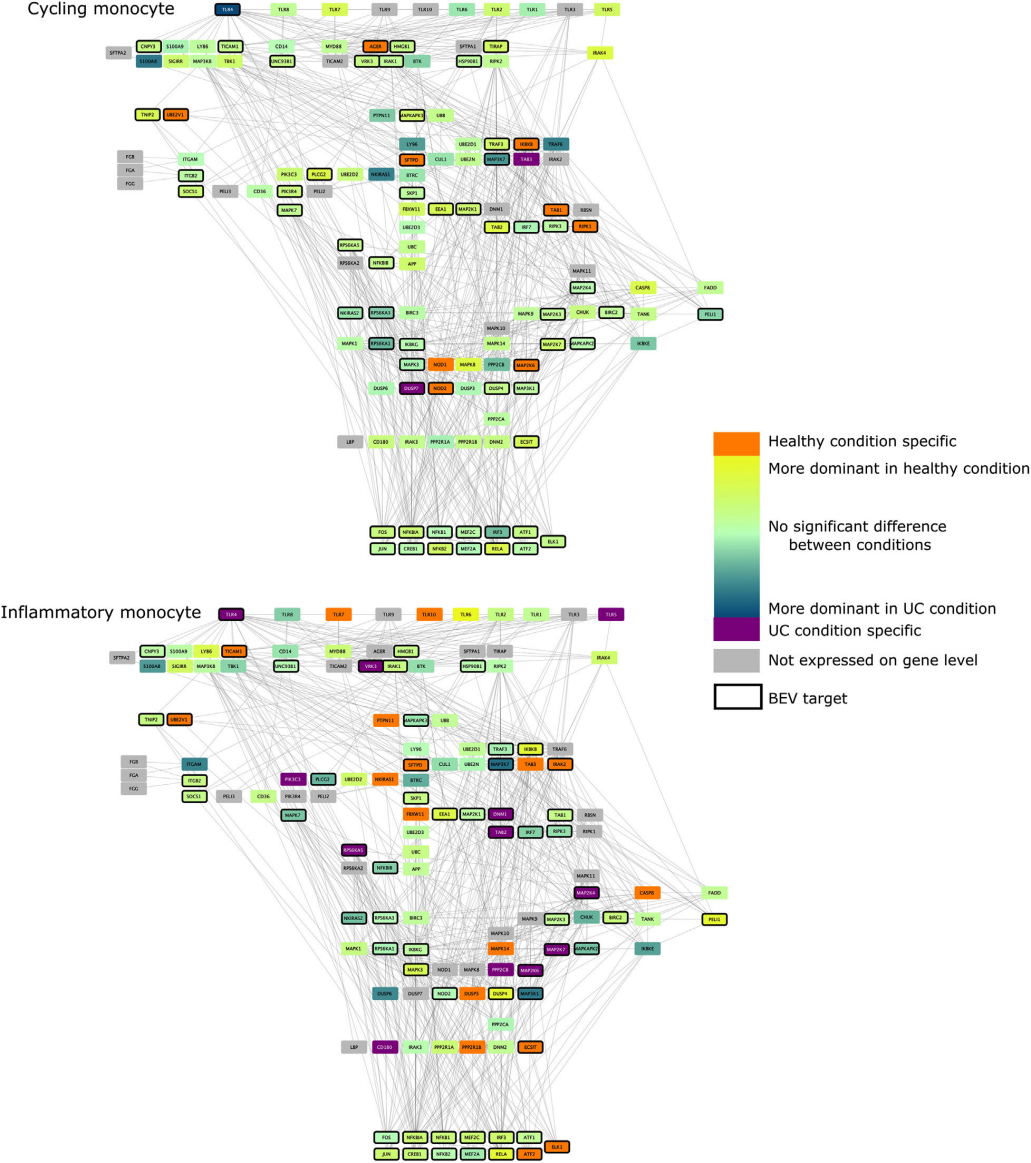
TLR pathway in monocytes. Edges between nodes represent protein‐protein interactions. Figures have been created with Cytoscape (Shannon et al., 2003)

**FIGURE 6:**
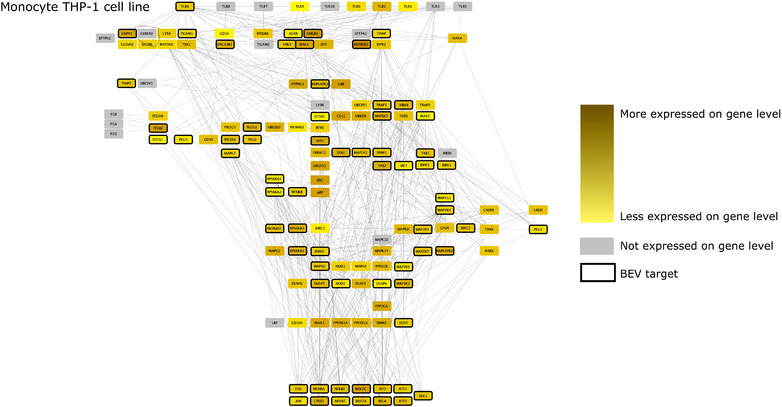
TLR pathway in THP‐1 monocytes (based on bulk transcriptomic datasets). Edges between nodes represent protein‐protein interactions. Figures have been created with Cytoscape (Shannon et al., 2003)

**FIGURE 7:**
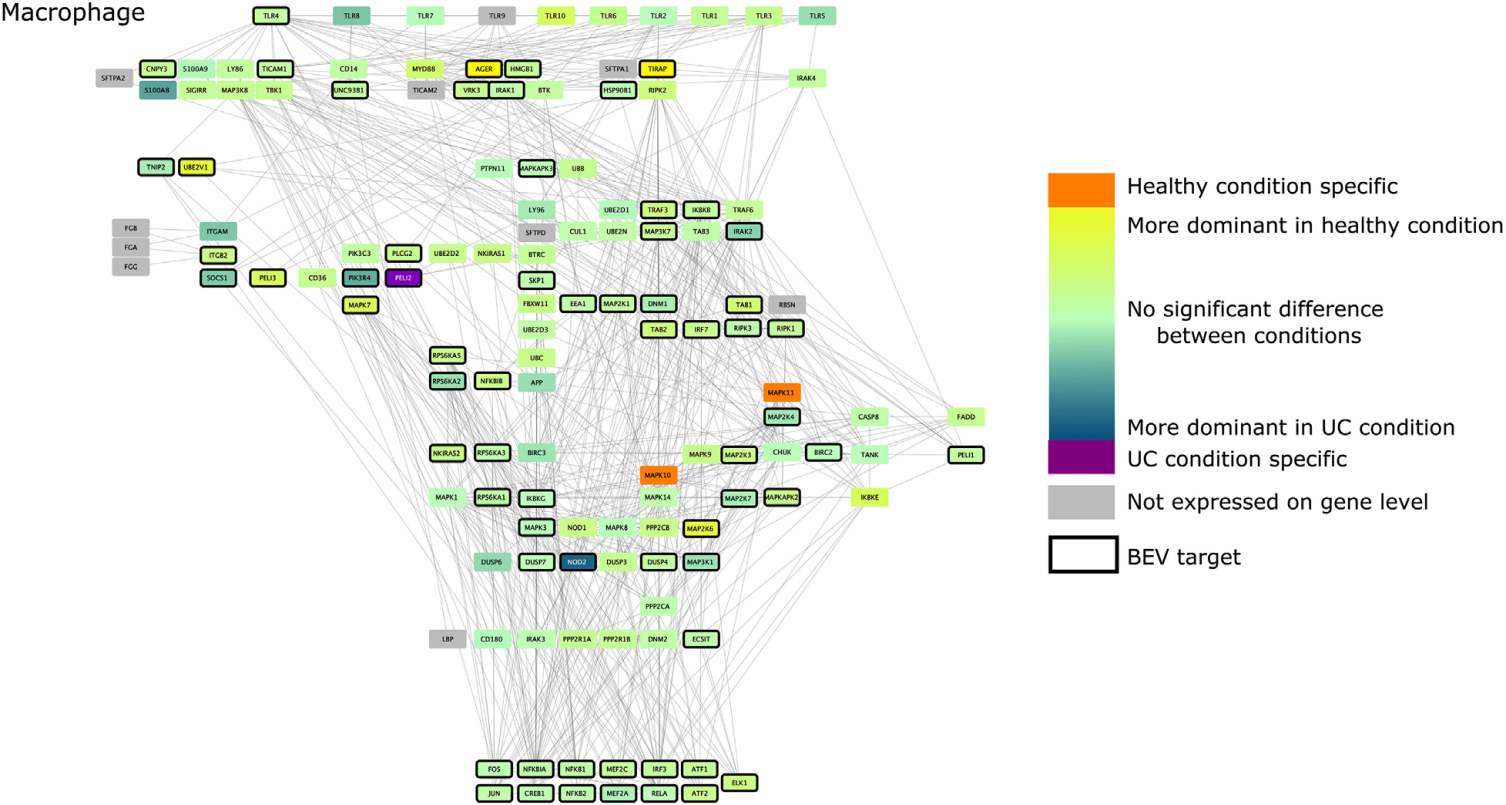
TLR pathway in macrophages. Edges between nodes represent protein‐protein interactions. Figures have been created with Cytoscape (Shannon et al., 2003)

As previous results established that Bt may alter immune pathways, we focused on the potential interactions between BEVs and TLR pathways. To do this, we created cell‐type and condition specific signalling networks for BEVs and TLR pathways based on the scRNAseq data. These networks revealed that whilst the expression of TLR pathway‐related transcription factors remained the same in both healthy and non‐inflamed UC conditions in all examined cell‐types, the level of TLR receptor expression was different amongst different immune cell‐types. Due to the cell‐type specific expression of different pathway members, BEV proteins established diverse interactions with immune cells (Figure 4, 5, 6, 7).

Analysis of TLR pathways demonstrated cell specificity, especially in monocytes and DC1 cells, with differences occurring mostly at the level of receptor proteins. The BEV‐targeted genes/proteins which show cell or condition specific expression may relate to the activation of different signalling pathways in healthy or UC. The network predictions also indicate that bacterial proteins can have intracellular immunomodulatory effects by binding to downstream elements of the TLR pathway (Figure 4, 5, 6, 7). The expression of genes encoding transcription factors (TFs) did not show divergence between healthy and diseased conditions.

Dendritic cell subsets (DC1–DC2) show diverse characteristics regarding expression of TLR pathway members with fewer pathway members being expressed in DC1s. Also, in DC1 cells under healthy conditions, a large number of TLR pathway members were expressed in a condition‐specific manner, including TLR1, 2, 3 and 7. In DC2 cells, three proteins were uniquely found in healthy (TLR3, MAPK7, and PP2R1B) and three in non‐inflamed UC (TAB3, DNM1, and PELI3) conditions. In addition, more TLR receptors (TLR1‐8, TLR10) were represented in DC2 cells compared to DC1 cells. However, a smaller number of differences were detected in the expression of TLR pathway members in DC2 cells compared to DC1 cells. While no receptor was targeted in DC1, TLR4 was identified as a potential BEV target in DC2 cells (Figure 4). These results raise an interesting issue regarding the DC subpopulation‐specific LOS mediated activation via TLR2/4 mediated signalling: DC1s are likely to not bind LOS in diseased condition due to the lack of *TLR4* expression and health‐related TLR2 expression. In contrast, *TLR2* and *TLR4* expressed in inflammation‐related DC2 in both healthy and UC conditions, enabling LOS mediated activation in both health and disease states.

In monocytes, the majority of TLR pathway members were expressed with signals being spread through diverse paths due to a few key signalling proteins being represented only in the healthy or diseased network. In terms of cycling monocytes, *TLR1, 2, 5, 6, 7, 8* were expressed at equivalent levels in both conditions, with *TLR4* expression strongly related to the diseased condition.

Amongst downstream signalling components, nine proteins were represented in the healthy state and two proteins in non‐inflamed UC with BEV proteins being able to bind most of them. In inflammatory monocytes several condition‐specific pathways were identified including TLR4 and TLR5 in non‐inflamed UC, and TLR7 and TLR10 pathways in the healthy state. The network shows a high number of condition‐specific proteins downstream (17 healthy and 12 UC specific proteins) (Figure 5). These results show that BEV proteins bind one TLR receptor (TLR4) which is expressed in both cell‐types but only in inflammation‐related monocytes in non‐inflamed UC.

We analysed bulk RNAseq datasets to verify the role of BEVs on the TLR4 pathway in THP‐1 monocytic cell line derived from human leukaemia (Tsuchiya et al., 1980). Results showed a more similar network to the output of the cycling monocyte scRNAseq data analysis. However, we found some differences in TLR pathways, revealing more potential for BEV‐interacting proteins (PELI2‐3, IRAK2, DNM1, RPS6K2, MAPK11) (Figure 6).

Based on the pipeline, macrophages depict no significant alteration in UC compared to the healthy state. While 9/10 receptors are potentially represented, TLR4 was the only candidate interacting with BEV proteins. MAPK10‐11 helped spread the signal in healthy cells, while PELI2 was expressed only in diseased macrophages (Figure 7).

### 3.4 Inhibition of TLR4 and TIRAP signalling abrogates BEV‐driven monocyte activation

**FIGURE 8:**
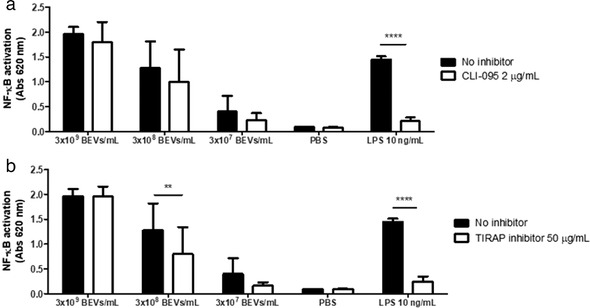
Inhibition of TLR4 and TIRAP signalling pathway abrogates THP1‐Blue cells activation by Bt BEVs. NF‐κB activation was assessed using different doses of BEVs in 5 × 10^5^ THP1‐Blue cells/ml in the presence or absence of the TLR4 inhibitor CLI‐095 (a) or TIRAP inhibitor (b) and by measuring absorbance at 620 nm after incubation with the colorimetric assay reagent Quanti‐Blue.LPS from *E. coli was used* as a positive control and PBS as a negative control. Data are presented as mean ± SD (*n* = 9). Significant differences were determined by using two‐way ANOVA followed by Bonferroni's multiple comparison post hoc test. ** (*P*  <  0.01), **** (*P*  <  0.0001)

Our pipeline identified TLR4 as the only receptor associated with BEVs in cycling monocytes, DC1 and macrophage cells. We therefore investigated the effects of BEVs on TLR4‐mediated activation of monocytes in BEV‐monocyte co‐cultures. Serial dilutions of Bt BEVs (3 × 10^9^–3 × 10^7^/ml) were cultured with THP1 monocytes expressing an NF‐kB reporter gene (THP1‐Blue). These experiments were carried out in the presence or absence of the TLR4 inhibitor, CLI‐095 (Ii et al., 2006; Kawamoto et al., 2008), which in pre‐optimisation experiments using *E. coli* derived LPs was shown to selectively inhibit TLR4 mediated activation of NF‐kB (data not shown). CLI‐095 achieved significant levels of inhibition of BEV‐mediated NF‐kB activation with the highest level of inhibition (∼37%) seen at the lower dose of BEVs (3 × 10^7^). By comparison, THP1‐Blue cells exposed to CLI‐095 in the absence of BEVs showed no significant inhibition (*P* > 0.05) of NF‐kB activation (Figure 8a). The inability to completely inhibit BEV‐induced THP‐1 activation by CLI‐095 suggests TLR4‐independent effects and pathways of BEVs induced NF‐kB‐activation. This potential is revealed in the TLR signalling network that identifies the BEV interacting downstream pathway components.

To substantiate and confirm the BEV‐TLR4 interaction in NF‐kB activation, we repeated the BEV‐THP‐1 co‐culture experiments using an inhibitor of TIRAP, which is an intracellular adaptor protein and component of the TLR4 and TLR2 signalling pathways. Pre‐incubation of THP1‐Blue cells with the TIRAP inhibitor prior to incubation with 3 × 10^8^ BEVs/ml demonstrated a significant (*P* < 0.01) reduction of NF‐kB activation (37.5%). At higher doses of BEVs, the TIRAP inhibitor was less effective at inhibiting NF‐kB activation (Figure 8b).

## 4 DISCUSSION

BEVs contain proteins capable of affecting the host immune system (Kuehn & Kesty, 2005). However, the molecular mechanisms of BEV‐mediated signalling in host cells are poorly understood. The recent availability of scRNAseq data facilitates the analysis of biological pathways at cell‐type specific resolution, which we utilised here to develop a computational workflow to identify the differential effects of BEV exposure on different populations of host immune cells.

Specifically, we examined proteins in BEVs generated by the major human commensal gut bacterium, Bt, which is a potential therapeutic agent in IBD (Delday et al., 2019). Hence, it is important to understand which, and how, specific cell‐types are affected by Bt BEVs. Considering gene expression profiles are different not only among cells but also in the same cells under different conditions, the possible protein‐protein interactions will vary between microbes and its host.

The computational pipeline combines single‐cell transcriptomics with network biology approaches to reconstruct the interactomes and model the effect of Bt BEVs on different immune cells. In particular, we used a publicly available human scRNAseq dataset to examine how Bt BEVs could potentially impact cycling monocytes, inflammatory monocytes, DC1s, DC2s, and macrophages in both the healthy, disease‐free colon and the non‐inflamed UC, diseased colon (Smillie et al., 2019). The output of the workflow highlighted that Bt BEVs have a large number of interactions with these immune cells. The majority of candidate interacting BEV proteins are catabolic enzymes with numerous non‐specific connections with our workflow highlighting bacterial proteins carrying PDZ domains. PDZ domains can assemble signalling complexes recognising a C‐terminal motif on the interacting protein and can change non‐specific PPIs to more specific interactions. The two main functions of PDZ domains are related to protein location determination and signalling, including cell‐cell communication (Harris & Lim, 2001; Lee & Zheng, 2010). Beside catabolic and PDZ domain‐containing BEV proteins, we identified microbial helicases targeting specifically the human polymerase protein PAPD5. Binding of helicases to polymerase proteins is critical to initiate leading‐strand DNA synthesis (Zhang et al., 2011). PAPD5 is also a well‐known negative regulator of miR‐21. Among the targets of this miRNA are genes involved in the immune responses and pathogenesis of autoimmune diseases, including IBD (Boele et al., 2014; Wang et al., 2016).

Despite the large overlap of connections, we identified in five types of immune cells unique functions triggered by Bt BEVs in the healthy and UC colon. For example, cell division is significantly enriched in cycling monocytes in the healthy state. In healthy conditions, bone marrow‐derived monocytes circulate in the blood and differentiate to macrophages in various tissues. Therefore, the proliferation of monocytes is required to maintain a pool of tissue‐specific monocytes and macrophages (Swirski et al., 2014). We also inferred that in UC, DNA repair activity might be influenced by BEV proteins interacting with cycling monocytes. Prior work has demonstrated that patients with UC have higher levels of mucosal oxidative DNA damage, even under non‐inflamed conditions, which increases with the duration and severity of disease (Aslan et al., 2011; Beltrán et al., 2010; D'incà et al., 2004; Dincer et al., 2007; Lih‐Brody et al., 1996). This is a potential explanation for the higher incidence of colorectal cancer in UC patients. Indeed, mice with chronic colitis that are deficient in a key DNA repair enzyme have increased susceptibility to developing colorectal carcinoma in response to oxidative stress (Liao et al., 2008). Our findings suggest Bt BEV proteins may play an important role in promoting DNA repair activity against oxidative DNA damage in cycling monocytes in patients with UC.

In inflammatory monocytes, BEV proteins upregulate apoptosis and the ERAD pathway in both healthy and UC states. Both these cellular processes are critical components of the unfolded protein response (UPR), which is important for resolving ER stress. Interestingly, our analysis also showed that BEVs influence ER‐stress response related processes in most immune cell types we studied. In UC, several risk variants affect genes involved in these pathways and together with environmental factors (such as intestinal microbial dysbiosis, metabolites and/or inflammatory cytokines), disrupt the UPR in intestinal epithelial cells. The resultant unabated ER stress has been shown to precipitate intestinal inflammation. However, in monocytes and macrophages higher levels of UPR transcripts have been found in DSS‐colitic mice compared to control mice, suggesting that the UPR may permit these cells to survive in the inflamed mucosal milieu of colitis (Jones et al., 2018). Thus, BEV proteins may help promote resolution of ER stress and maintain the survival of inflammatory monocytes, macrophages, and other immune cells by upregulating key components of the UPR.

Dendritic cells are key antigen presenting cells and play important roles in innate and adaptive immunity including responses to microbial pathogens. Interactions between DCs and BEVs can direct inflammation in the gut (Durant et al., 2020). The microbiome can promote the differentiation of immature DCs into diverse subpopulations therefore maintaining immune homeostasis (Stagg, 2003).

We focused on the effects of BEVs on TLR pathways examining different activities and outcomes of the pathways. A prior study in mice indicates that Bt is capable of binding TLR4 (Coats et al., 2016). Here we discovered that Bt BEVs interact with TLR4 in a cell‐type and condition specific manner. Of note, TLR4 expression is upregulated in the inflamed colonic mucosa of UC patients at both mRNA and protein levels (Hug et al., 2018; Levin & Shibolet, 2008).

Our pipeline was used to investigate if Bt BEV proteins might trigger immune response not only extracellularly via surface receptor interactions, but also by interacting with intracellular proteins. Based on our Bt BEV‐human PPI network, a bacterial carboxyl‐terminal protease (BT_2239) is predicted to bind TLR4. There is however no evidence as to how this enzyme affects TLR4 activation, although in chickens TLR15 can be triggered by microbial proteases (De Zoete et al., 2011). The domain‐motif prediction approach of our pipeline provides more structural details about host‐microbe interactions: BT_2239 interacts with TLR4 by a PDZ domain which catch a short motif —between 833 and 839 amino acid positions — at the end of the host protein's intracellular TIR domain. This suggests a possible intracellular BEV‐TLR4 interaction separate or in addition to the extracellular LOS‐TLR4 interactions. Evidence in support of this proposal was obtained using the TLR4 inhibitor CLI‐095 which binds to and inhibits interactions with the intracellular domains of TLR4 and abrogated BEV‐mediated NF‐kB activation of THP‐1 monocytes. Further confirmation of the nature of BEV‐TLR4 interactions was obtained by blocking the TLR2/4 adaptor protein TIRAP that similarly inhibited BEV‐mediated NF‐kB activation of THP‐1 monocytes. Of note, *in silico* analysis revealed cell and condition specific expression of TIRAP. It is expressed in both healthy and disease states in cycling monocytes, DC2s and macrophages. Nevertheless, the adaptor protein does not appear to be expressed by inflammatory monocytes and only under healthy conditions in DC1s. PPI prediction revealed 19 BEV proteins which may interact with the TIRAP protein through diverse domain‐motif interactions. The differential expression and the high number of interacting bacterial proteins highlights a potentially important role of TIRAP in BEV‐related regulation of inflammation that could be explored further as a potential therapeutic target in IBD. The co‐localization of Bt BEVs with various intracellular compartments and in particular, the nucleus, of intestinal epithelial cells that have acquired BEVs (Jones et al., 2020) demonstrates the feasibility of Bt BEVs interactions with various cytoplasmic constituents of host cells.

Whilst providing new and potentially important insights into BEV‐host immune cell interactomes our pipeline is limited to one available scRNAseq dataset that describes gene expression in healthy and non‐inflamed UC colonic mucosal cells. Some expressed genes could be missed with the 10X single‐cell transcriptomics approach, and we also do not have corresponding protein levels (or their activities) in the cells of interest. In inferring a microbe‐host PPI network, we assumed that all genes were translated to functional proteins regardless of post‐transcriptional modifications that could affect protein abundance. Regarding the PPI predictions for the microbe‐host interactions we used a limited list of domain‐motif interactions from the ELM database and also only eukaryotic Pfam domains are represented in the analysis which means prokaryotic‐specific domains (e.g., S41 proteases) are missing from the network analysis. Finally, our workflow cannot predict the activation or inhibitory effects of BEV proteins, but only whether they act on a particular receptor and pathway. Further investigations are needed to establish the binding mechanism and impact of for example, the BEV carboxy‐peptidase on host TLR4 receptors. Despite these limitations, our pipeline provides a deeper insight into the effect of BEV proteins on host immunity at the protein level and shows the importance of condition and cell specificity. In addition to predicting the affected host processes supported by the literature our computational pipeline also identified new targets for experimental validation.

## 5 CONCLUSION

We have developed a computational pipeline that predicts both the cell and condition specific effects of Bt BEV proteins on key host immune cell populations. Focusing on the inflammation‐related TLR pathway, which plays a role in IBD pathogenesis, our workflow highlighted the importance of single‐cell based analysis identifying differences in TLR4 receptor expression in diverse DC subpopulations. The current pipeline offers potentially interesting connection points and detailed mechanistic insight — using structural information about proteins — into the relationship between Bt and host immune cells that will aid in understanding how BEVs and their protein cargo may be of therapeutic value in IBD.

## CONFLICT OF INTEREST

The authors report no conflict of interest.

## Supporting information

Supporting information.

Supporting information.

Supporting information.

Supporting information.

Supporting information.

Supporting information.

Supporting information.
