# Supplementary figures and images for: Extracellular vesicles produced by the human commensal gut bacterium Bacteroides thetaiotaomicron affect host immune pathways in a cell‐type specific manner that are altered in inflammatory bowel disease
Source: J Extracell Vesicles. 2022 Jan 22;11(1):e12189. doi: 10.1002/jev2.12189 (PMC8783345; doi:10.1002/jev2.12189)

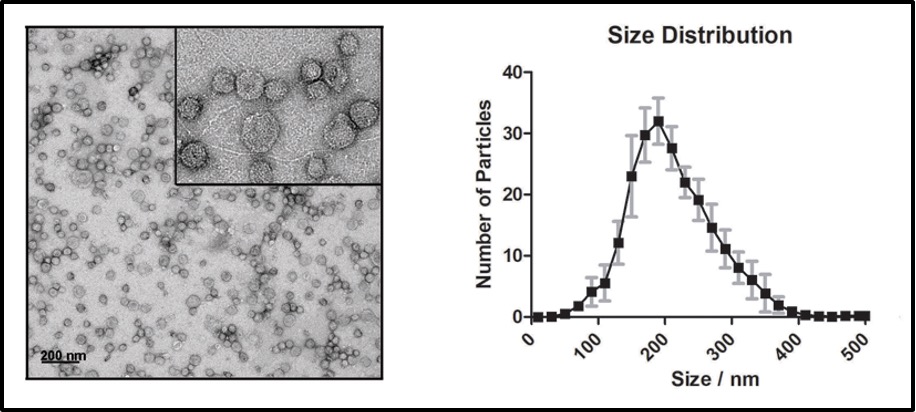

Supplement: Supplementary file 1 — Supporting information. [file JEV2-11-e12189-s007.jpg]

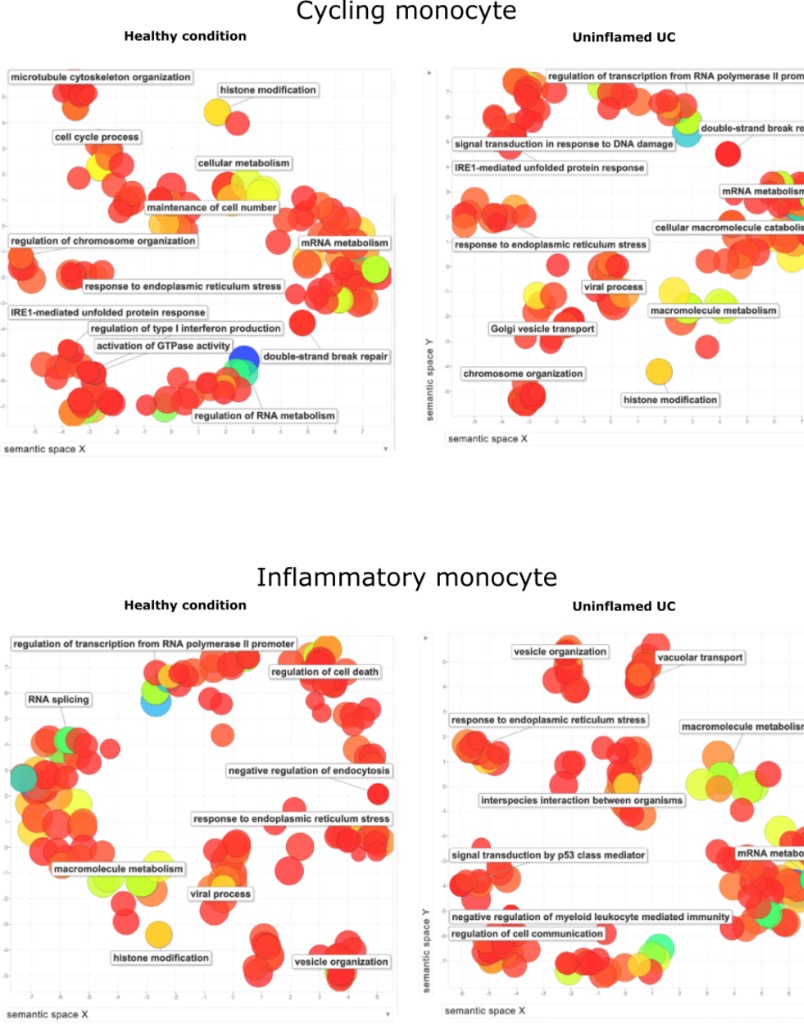

Supplement: Supplementary file 2 — Supporting information. [file JEV2-11-e12189-s001.jpg]

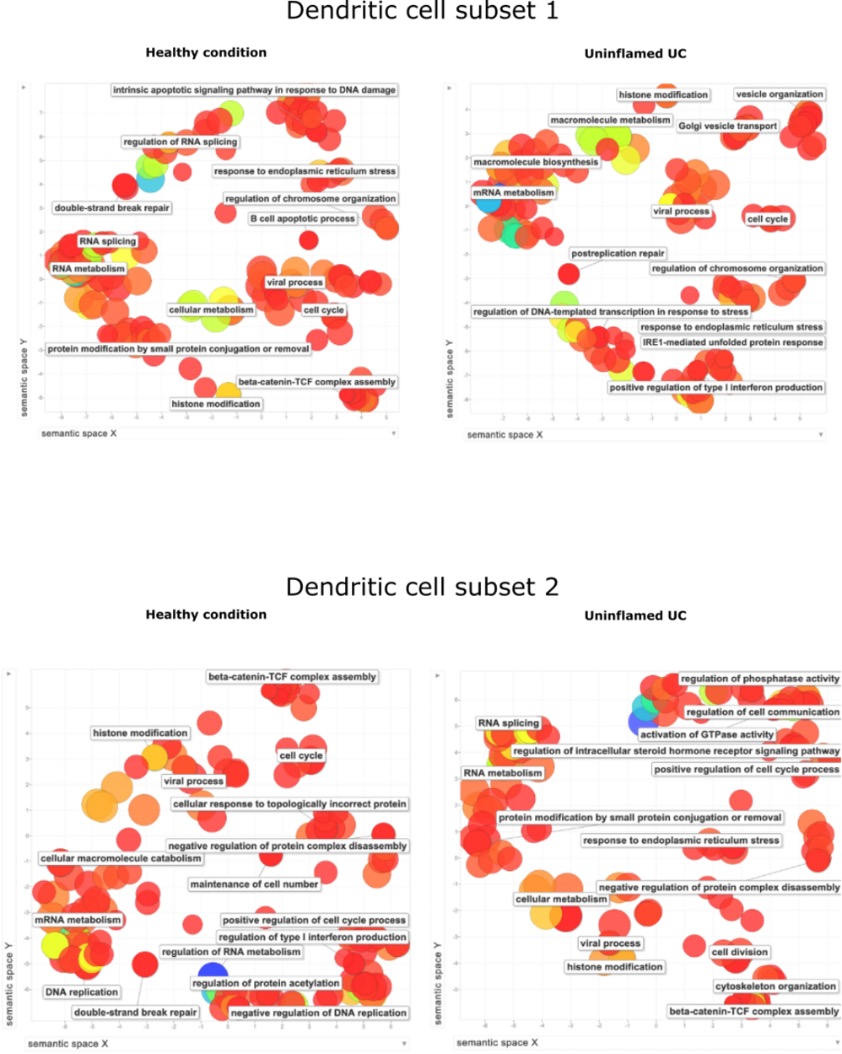

Supplement: Supplementary file 3 — Supporting information. [file JEV2-11-e12189-s004.jpg]

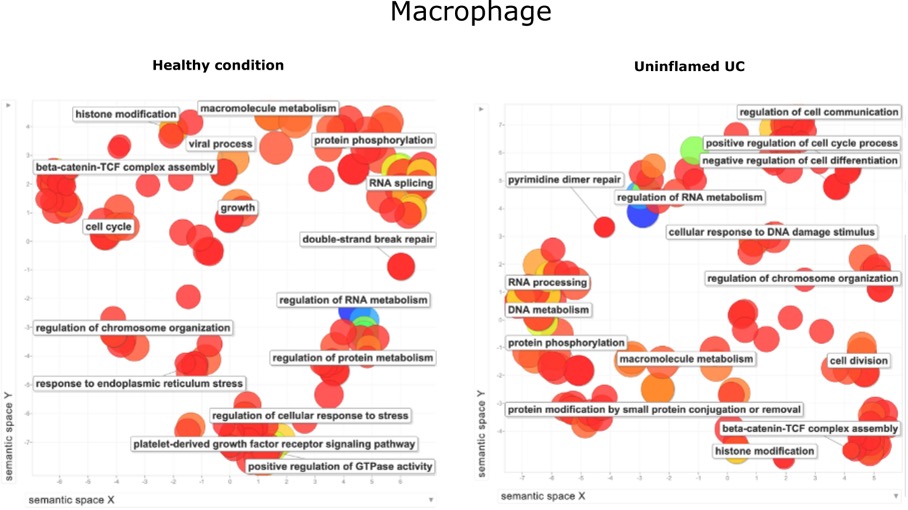

Supplement: Supplementary file 4 — Supporting information. [file JEV2-11-e12189-s006.jpg]
